# Correction: Two Ln-based metal–organic frameworks based on the 5-(1*H*-1,2,4-triazol-1-yl)-1,3-benzenedicarboxylic acid ligand: syntheses, structures, and photocatalytic properties

**DOI:** 10.1039/d0ra90125c

**Published:** 2021-01-04

**Authors:** Fei Yuan, Chunmei Yuan, Baoyue Cao, Youying Di, Shumin Wang, Mingbao Liu, Abhinav Kumar, Chuncheng Shi, Mohd. Muddassir

**Affiliations:** Shaanxi Key Laboratory of Comprehensive Utilization of Tailings Resources, College of Chemical Engineering and Modern Materials, Shangluo University Shangluo 726000 China slyuanf@126.com happywshm@126.com; Department of Chemistry, Faculty of Science, University of Lucknow Lucknow 226007 India abhinavmarshal@gmail.com; Department of Pharmacy, School of Medicine, Xi’an International University Xi’an 710077 Shaanxi China shichch66xaiuedu@163.com; Department of Chemistry, College of Sciences, King Saud University Riyadh 11451 Saudi Arabia

## Abstract

Correction for ‘Two Ln-based metal–organic frameworks based on the 5-(1*H*-1,2,4-triazol-1-yl)-1,3-benzenedicarboxylic acid ligand: syntheses, structures, and photocatalytic properties’ by Fei Yuan *et al.*, *RSC Adv.*, 2020, **10**, 39771–39778, DOI: 10.1039/D0RA07159E.

The authors regret that in the Graphical Abstract of the original article, incorrect figures were included. The Graphical Abstract online has been replaced with an updated version.

The Royal Society of Chemistry apologises for these errors and any consequent inconvenience to authors and readers.

## Supplementary Material

